# NK/T-cell lymphoma with rash and peripheral neuropathy as the first manifestation: a case report and literature review

**DOI:** 10.1186/s13000-023-01286-z

**Published:** 2023-01-10

**Authors:** Zizhu Tian, Jing Tian, Jiafen Liao

**Affiliations:** 1 Department of Hematology, the Second Xiangya Hospital, Central South University, Changsha, 410011 Hunan China; 2 Department of Rheumatology and Immunology, the Second Xiangya Hospital, Central South University, Changsha, 410011 Hunan China

**Keywords:** ENKTCL-NT, Peripheral neuropathy, CD30 positive, EBV infection, Hemophagocytic syndrome

## Abstract

**Background:**

Extranodal NK/T-cell lymphoma, nasal type (ENKTCL-NT), is a rare, aggressive subtype of non-Hodgkin lymphoma, and it usually presents as a destructive sinus mass that may attend epistaxis. However, ENKTCL-NT with the manifestation of peripheral neuropathy is pretty unusual.

**Case presentation:**

A 17-year-old Chinese patient presented with peripheral neuropathy and positive auto-antibodies. However, she failed methylprednisolone pulse therapy and developed hemophagocytic syndrome. The diagnosis of CD30-positive primary cutaneous ENKTCL-NT was confirmed by pathological biopsy. Her disease was brought under control after five cycles of chemotherapy.

**Conclusions:**

The report findings are helpful in the differential diagnosis of peripheral neuropathy and autoimmune disease. We should be alert for the development of ENKTCL-NT when the rash and peripheral neuropathy are the first symptoms and are accompanied by Epstein-Barr virus (EBV) infection.

## Introduction

Extranodal NK/T-cell lymphoma, nasal type (ENKTCL-NT), is a highly aggressive subtype of non-Hodgkin’s lymphoma (NHL) and is often associated with Epstein-Barr virus (EBV) infection. ENKTCL-NT frequently involves the nose and upper aerodigestive tract (80%), with a few manifestations in external sites (20%) such as skin, gastrointestinal tract, testis, and salivary glands [1]. Adolescent ENKTCL with peripheral nerve involvement is even rarer and more challenging to diagnose. Here, we report a case of primary cutaneous ENKTCL-NT associated with peripheral neuropathy onset in a Chinese adolescent patient.

## Case report

A 17-year-old Chinese adolescent female presented with two sudden red raised rashes on her left calf in December 2021 without pain, itching, or ulceration, which gradually crusted over and exfoliated to form hyperpigmented plaques. Numbness, pain, and weakness in both hands occurred in February 2022. And due to worsening symptoms, she presented to a local hospital neurology department in March 2022. A peripheral blood test showed a series of positive auto-antibodies, including anti-nuclear antibody (ANA) titer of 1:80, anti-RNP, anti-Sulfatide, and anti-GM3 antibody IgG. Electromyography showed partial peripheral nervous system damage in both upper extremities. Enhanced magnetic resonance imaging (MRI) of both hands demonstrated edema of interosseous muscles, pinky-to-metacarpal muscles, and pinky flexors in both hands. In addition, abnormalities in the cerebrospinal fluid examination, as well as MRI and angiography of her head, did not reveal any abnormalities. She was diagnosed with connective tissue disease and secondary peripheral neuropathy. After methylprednisolone pulse therapy (500 mg/day × 3 days), the feeling of weakness in both hands gradually improved, but the numbness was the same as before. At the end of March 2022, she grew three red rashes on her left leg, which showed the same characteristics as those previously occurred in the left calf. The patient had a left-sided facial rash with ulceration in November 2013 when she was eight. Skin biopsy revealed a facial lymphocytic infiltrate, and serum auto-antibodies revealed positive anti-RNP (+++); the patient was subsequently diagnosed with granuloma annulare. She was treated with hydroxychloroquine and thalidomide for 4 months, and the rash subsided, with several follow-up tests turning antibody negative and no recurrence for 3 years. There was no history of mosquito bites or drug abuse.

**Fig. 1:**
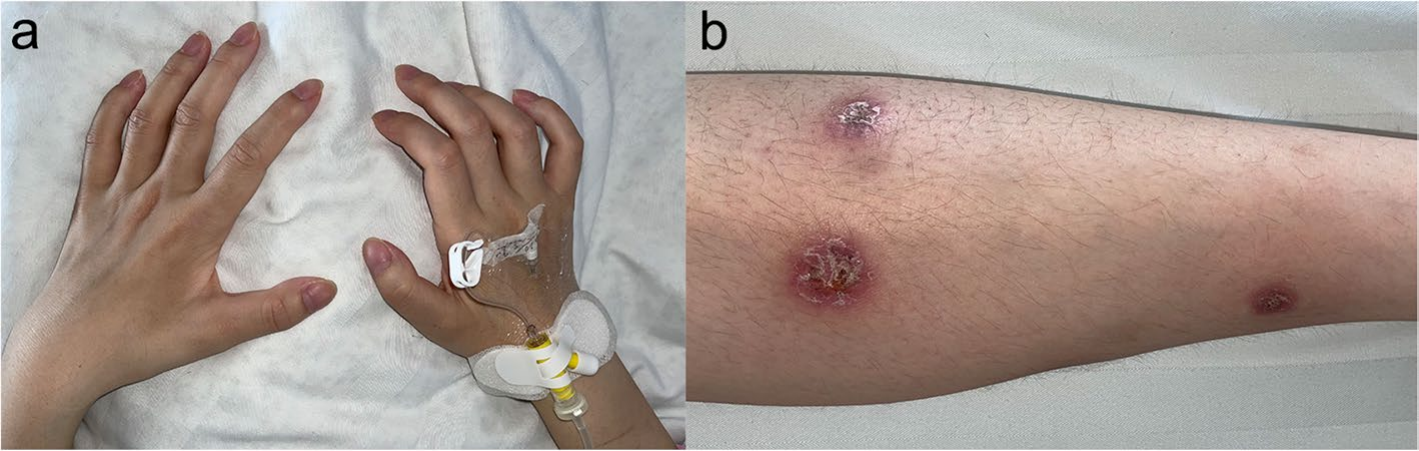
**a** Muscle atrophy of both hands at admission **b** Rash on left leg at admission, crusted over

For treatment, she was admitted to the department of Immunology and Rheumatology of our hospital in May 2022. Physical examination showed a temperature of 36.6 °C, pulse rate of 104 bpm, respiratory rate of 20 bpm, and blood pressure of 104/73 mmHg. The patient had atrophy of both hands **(**Fig. 1a**)**, grade 4 distal muscle strength of both upper limbs, and reduced superficial sensation in the dorsum of the hands. Two hyperpigmented plaques of approximately 1 × 2 cm in size and three crusted rashes of about 1 × 1.5 cm in length, all hard in texture, were visible on her left lower leg **(**Fig. 1b**)**. Superficial lymph nodes throughout the body, liver, and spleen were not palpated.

**Fig. 2:**
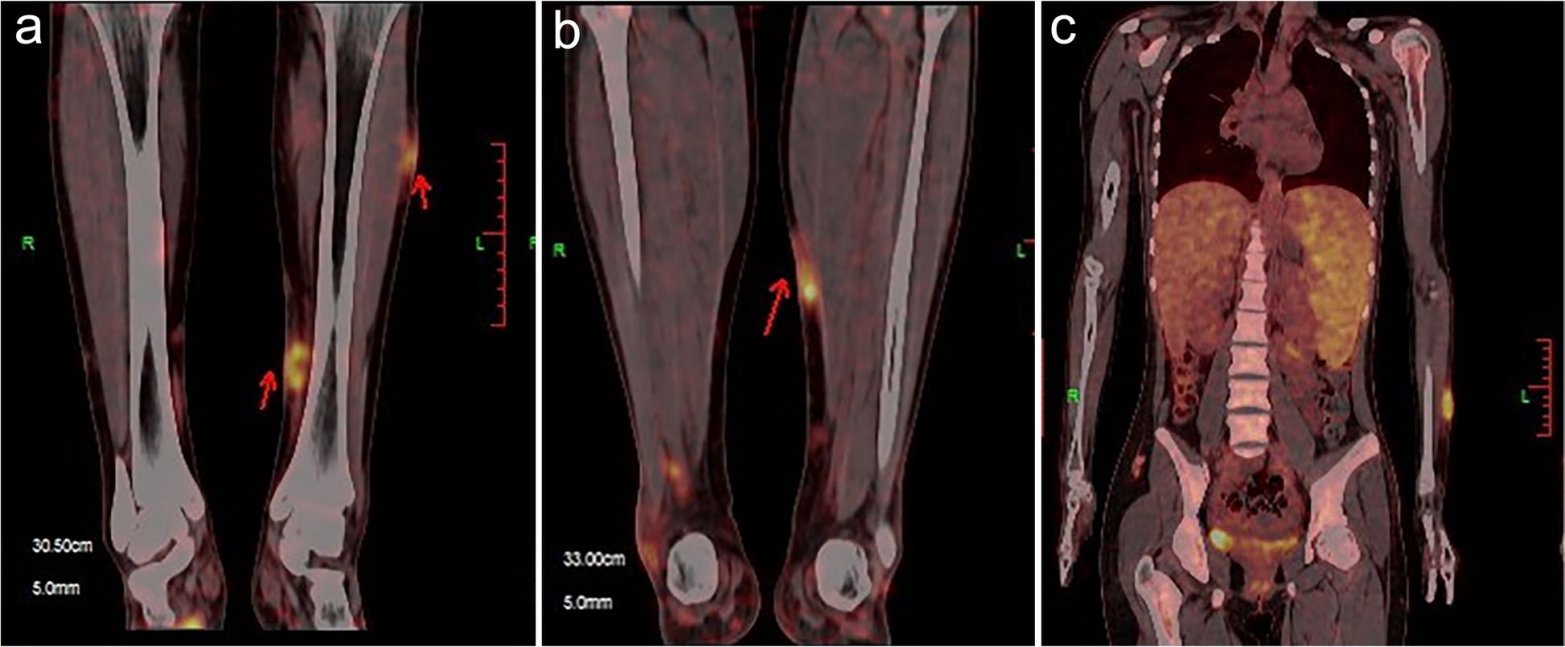
After completing a biopsy of the entire nodule in the right arm of the patient, PET-CT showed increased FDG metabolism in a-b) the Bilateral lower extremities and feet subcutaneous, the right medial-posterior knee muscle group, the left posterior muscle group, and c) the left forearm and spleen, with the highest located at the right knee muscle group and a SUVmax of 8.6

Laboratory investigations on this admission showed a complete blood count suggesting leukocyte count 2.63 × 10^9^/L (39.9% neutrophils), hemoglobin 109 g/L, lactate dehydrogenase (LDH) 267.4 U/L, EBV-DNA 1160copies/ml. Auto-antibodies such as extractable nuclear antigen, ANA, anti-neutrophil cytoplasmic antibodies, rheumatoid factor, anti-Sulfatide antibody IgG, and anti-GM3 antibody IgG were negative. HIV, viral hepatitis, and tuberculosis infection were excluded, and no abnormal tumor markers were observed. Bone marrow smear and biopsy, T cell receptor (TCR) γ gene rearrangement, and Ig gene rearrangement did not reveal any abnormalities. Nasopharyngoscopy showed residual adenoids and chronic pharyngitis. Electromyography showed symmetrical and multiple chronic electrophysiological damages of peripheral neurogenic origin in the upper and lower extremities; proximal nerve root demyelination damage, distal peripheral nerve fiber demyelination, and mixed axonal damage was apparent; Bilateral short thumb extensors and little finger extensors were found to have significant denervation potential, suggesting active damage. Positron emission tomography (PET-CT) scan showed increased fluorodeoxyglucose (FDG) metabolism in the left forearm, both lower extremities, and subcutaneously in the feet, at the medial-posterior muscle group in the right knee, and the left posterior muscle group, bilateral groin, and spleen, with the highest located at the right knee muscle group and a SUVmax of 8.6 (Fig. 2).

**Fig. 3:**
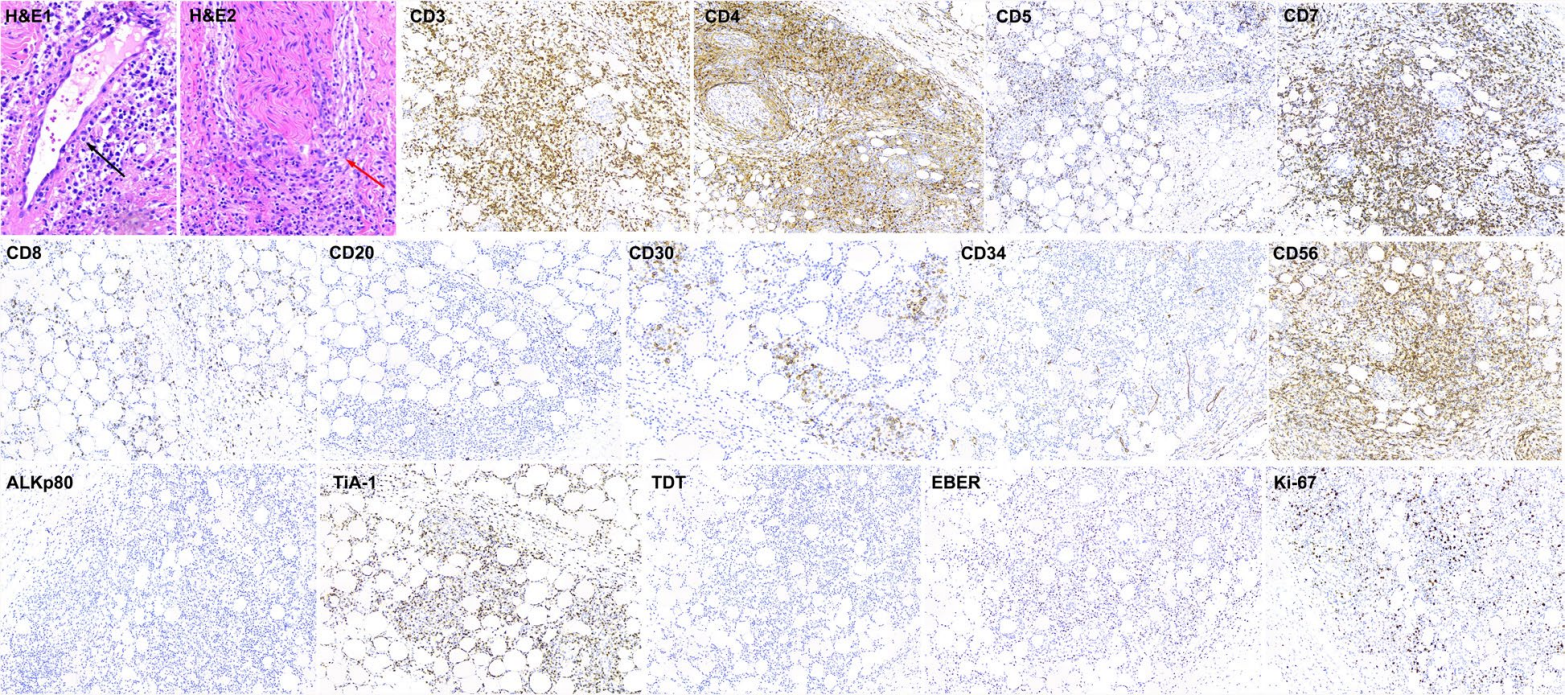
Immunohistochemical manifestation of the right forearm nodule. H&E stain(× 300) shows focal or sheet-like infiltration of heterogeneous lymphocytes with medium-sized cells and visible nuclear fragmentation and fission, in addition to cancer cells invading blood vessels (H&E1, black arrow) and nerves (H&E2, red arrow). Positive for CD3, CD7, CD56 and TiA-1, weakly positive for CD4, 10% positive for CD30 hotspot area(× 200), negative for CD5, CD8, CD20, CD34, ALKp80 and TDT, 30% positive for Ki-67, positive for EBER in situ hybridization (except H&E and CD30, others × 100)

On the first day after admission, the patient presented with a new subcutaneous nodule on each upper extremity and left lower extremity, each about 1 × 1 cm in size, with a hard texture and clear borders. A skin biopsy was subsequently scheduled. Partial excisional biopsy of the left arm nodes showed focal or sheet-like infiltration of heterogeneous lymphocytes in the dermis and subcutaneous adipose tissue, with medium-sized cells, round or irregular nuclei, moderate cytoplasm, visible nuclear fragmentation, and nuclear fission. Immunohistochemistry (IHC) showed positivity staining for CD3, CD7, CD56 and TiA-1, weak positive for CD4, CD8, CD30 (3%+),and negative for CD20, ALKp80. The positive rate of the Ki-67 hot spot region in the cells was 50%. Monoclonal rearrangement of T lymphocyte genes was negative. However, the fever began appearing on the 6th day after admission, with the highest body temperature of 40 degrees Celsius. Lactate dehydrogenase, ferritin, and triglycerides increased progressively; and leukocytes, hemoglobin, platelets, and fibrinogen showed a downward trend; soluble CD25 (SIL-2R) value of 2263 U/ml (greater than three times the upper limit of normal) and NK cell activity of 3.04%, all suggesting the occurrence of hemophagocytic syndrome. Another skin biopsy was scheduled. A whole excisional biopsy was taken of the neoplastic node in the right arm. The cell morphology was similar to that of the left arm, and cancer cells were found to invade nerves and blood vessels (Fig. 3). IHC of the right arm node revealed positive staining for CD3, CD7, CD56, and TiA-1, weakly positive for CD4, and negative for CD5, CD8, and CD20. CD30 hot spot showed a 10% positive rate, and the Ki-67 positive rate was 30% (Fig. 3). Both biopsies were positive for EBV-encoded small nuclear RNA (EBER) in situ hybridization and negative for monoclonal rearrangement of T lymphocyte genes. The immunophenotypes were all consistent with the diagnosis of ENKTCL-NT.

The patient was finally diagnosed with ENKTCL-NT (stage IV), secondary peripheral neuropathy, and hemophagocytic syndrome. Due to the rapid deterioration of the patient’s condition and the late appearance of the second pathological biopsy results, chemotherapy was first administered for the hemophagocytic syndrome using HLH-94 protocol in combination with the ruxolitinib, followed by the P-Gemox regimen for the primary disease. Her temperature dropped to normal. A follow-up PET-CT at the end of the 4th chemotherapy cycle showed a marked decrease in radioactive uptake in the left forearm, both lower limbs, and both feet subcutaneously (current SUVmax 1.27, previous SUVmax 6.81), bilateral groin (current SUVmax 1.06, previous SUVmax 3.84), and spleen (current SUVmax 2.40, previous SUVmax 3.9) compared to the previous, with Deauville score 1–2. The previously increased FDG metabolism in the right posterior infrapatellar muscle group and left posterior muscle group nodes was not shown, which indicated partial remission. Until October 2022, the patient had completed five cycles of P-Gemox chemotherapy. The generalized rash had subsided mainly, with only a rash of approximately 0.4 × 1.2 cm on the left calf visible on the exterior. For financial reasons, the patient has not yet been considered for hematopoietic stem cell transplantation.

## Discussion

ENKTCL-NT is an aggressive lymphoma associated with EBV infection that originates from mature natural killer cells. It has a high incidence in East Asia and Latin America, accounting for approximately 10% of NHL; however, it accounts for less than 1.5% of NHL in Europe and the United States. This variability is associated with genetic factors in Asians. The median age of patients with ENKTCL-NT is 46–60 years, mainly seen in males [2]. Thus, it is rare in children and adolescents, especially in females. Histopathological biopsies reveal medium-sized cells with pleomorphic, vascular-centered infiltration with vascular injury and vascular wall necrosis, usually with nuclear rupture and apoptotic vesicles. Immunophenotype showed CD3 and cytotoxic granules, CD56, CD43, and CD45RO, were typically positive. T cell lineage expressed CD5 and CD8, but TCR, CD4, CD16, and CD57 were rarely expressed. In situ hybridization, EBV expression was positive. The patient we report is the first case of EBV infection-associated CD30-positive ENKTCL-NT with the concomitant invasion of the peripheral nervous system.

Diagnosing ENKTCL in children and adolescents is challenging. It must be distinguished from other EBV infection-related diseases, such as infectious mononucleosis (IM) and chronic active EBV infection (CAEBV), which are difficult to identify based on morphology and immunophenotype alone. IM is caused by a transient proliferation of B cells and an overreaction of cytotoxic T cells due to EBV infection, which manifests as fever, sore throat, and generalized lymph node enlargement. A minority of IM may evolve into CAEBV, which presents with recurrent IM-like symptoms, including fever, enlarged liver, spleen, and lymph nodes, liver failure, and high EBV DNA load in peripheral blood, which in turn may lead to the hemophagocytic syndrome [3]. In contrast, ENKTCL usually starts as a localized and progressive mass. Thus, a combination of history, clinical presentation, and EBV infection is needed to differentiate. The patient initially had rashes and peripheral neuropathy, normal liver function on admission, no palpable enlargement of the liver or superficial lymph nodes, and a relatively low starting EBV copy number that gradually increased with disease progression, all inconsistent with the typical presentation of IM and CAEBV. With the recurrent rash of the patient and two pathological biopsies suggestive of ENKTCL, we consider the occurrence of hemophagocytic syndrome in the present case to be probably due to the interaction of EBV infection and tumor.

The case was considered a connective tissue disease at the initial diagnosis due to a series of positive autoimmune antibodies. According to the Kahn criteria [4], the patient had a low titer of anti-RNP antibodies, no finger swelling, synovitis, myositis, or Raynaud’s phenomenon, and all of the autoimmune antibodies turned negative when the patient was seen again 2 months later, so the diagnosis of mixed connective tissue disease was not supported. Meanwhile, the patient’s peripheral nerve demyelination lesions should be distinguished from Guillain-Barré syndrome, an acute immune-mediated inflammatory peripheral neuropathy in which the cerebrospinal fluid often shows protein-cell separation, progressive worsening of the neuropathy, with a peak within 2 weeks, and respiratory muscle weakness in severe cases. The patient we report mainly presented with bilateral upper extremity peripheral nerve demyelination lesions without progressive progression and spread, no history of antecedent infection, and no abnormalities in cerebrospinal fluid tests. Accordingly, Guillain-Barré syndrome was not established. Reviewing the patient’s history, the development of ENKTCL may cause autoimmune system disorders and peripheral neuropathy, but this is rare, and B-cell NHL is the dominant type involving peripheral nerves [5].

Based on our review of the English literature (Table 1), only 4 cases of ENKTCL-NT invasion of peripheral nerves have been reported [6–9]. These patients were from different countries. 75% were female (3/4), and their ages ranged from 20 to 59. Two started with peripheral neuropathy as the first symptom [6, 7]. Another two had a history of NK/T-cell lymphoma and were in remission before they presented with peripheral nerve involvement [8, 9]. Another finding was that chemotherapy seems to be a better choice over radiotherapy in ENKTCL-NT. The four patients who received chemotherapy, with or without steroids (including our patient), were able to observe disease remission, including complete remission (CR) in three patients [6, 8, 9] and partial remission (PR) in one patient (our case). However, patients treated with radiation alone had poor results [7].

**Table 1:** Clinical characteristics of patients with ENKTCL-NT combined with peripheral neuropathy

Authors, year	Age/Sex	Country	Initial symptoms	Nerve involvement site	Primary site of ENKTCL-NT	Treatment	Therapeutic effect	Duration of Follow-up (months)	Survival Status
Wills A J, et al., 2008 [6]	29/F	UK	Numbness and weakness of lower limbs and face	Facial and lumbar nerve	Ovarian adnexa	Steroids；chemotherapy	Complete remission	15	Survival
Morita M,et al., 2009 [7]	67/M	Japan	Leg pain, bladder and bowel dysfunction	Cauda Equina	Cauda Equina	Radiotherapy	Disease progression	14	Died
Agrawal S, et al., 2013 [8]	57/F	Singapore	Swelling and numbness in the right forearm	Ulnar nerve	NA	Chemotherapy	Complete remission	12	Survival
Aynardi M,et al., 2018 [9]	59/F	USA	Pain and numbness in right ankle with ulcer	Posterior tibial nerve	Nasal cavity	Radiotherapy+ Chemotherapy	Complete remission	12	Survival
Our case, 2022	17/F	China	Rash， Numbness, and weakness in both hands	Peripheral nerves of upper and lower extremities	Skin	Steroids；chemotherapy	Partial remission	5	Survival

*NA* Data not available

CD30, a member of the tumor necrosis factor receptor family, is characteristically expressed in classic Hodgkin’s lymphoma (HL) and anaplastic large cell lymphoma (ALCL), and a monomethyl auristatin E-conjugated anti-CD30 antibody (Brentuximab Vedotin) has been approved for the treatment of relapsed or refractory HL and ALCL [10, 11]. A retrospective study found that CD30 expression was detectable in 57% of patients with extranodal peripheral T-cell lymphomas (PTCLs) at a 5% threshold, suggesting that CD30-targeted therapy may be an effective treatment for PTCLs [12]. Although our patient is at an advanced stage of the disease, targeted therapies can be tried to prolong survival based on CD30 expression, if necessary.

In summary, we report a case of an adolescent patient with CD30-positive primary cutaneous ENKTCL-NT, secondary peripheral neuropathy, and hemophagocytic syndrome requiring differentiation from EBV infection-related disease, connective tissue disease, and Guillain-Barré syndrome. When encountering patients with unexplained peripheral neuropathy as the first symptom, along with EBV infection, the development of ENKTCL-NT should be alerted.

## Data Availability

The datasets used and/or analysed during the current study are available from the corresponding author on reasonable request.

## Abbreviations

ENKTCL-NT: Extranodal NK/T-cell lymphoma, nasal type
NHL: Non-Hodgkin’s lymphoma
EBV: Epstein-Barr virus
ANA: Anti-nuclear antibody
MRI: Magnetic resonance imaging
LDH: Lactate dehydrogenase
TCR: T cell receptor
PET-CT: Positron emission tomography
FDG: Fluorodeoxyglucose
EBER: EBV encoded small nuclear RNA
IM: Infectious mononucleosis.
CAEBV: Chronic active EBV infection
CR: Complete remission
PR: Partial remission
HL: Hodgkin’s lymphoma
ALCL: Anaplastic large cell lymphoma
PTCL: Peripheral T-cell lymphoma
